# Amelioration of Ambient Particulate Matter (PM_2.5_)-Induced Lung Injury in Rats by Aerobic Exercise Training

**DOI:** 10.3389/fphys.2021.731594

**Published:** 2021-10-26

**Authors:** Fei Qin, Zhengzheng Fan, Minxiao Xu, Zhongwei Wang, Yanan Dong, Chaoyi Qu, Shuqiang Cui, Lina Zhao, Jiexiu Zhao

**Affiliations:** ^1^Sport Biological Center, China Institute of Sport Science, Beijing, China; ^2^School of Physical Education, Jinan University, Guangzhou, China; ^3^Institute of Physical Education and Training, Capital University of Physical Education and Sports, Beijing, China; ^4^Athletic Sports Research Lab, Beijing Institute of Sports Science, Beijing, China

**Keywords:** aerobic interval training, ambient particulate matter, lung injury, inflammation, p38–COX_2_–PGE_2_ pathways

## Abstract

Ambient particulate matter (PM_2.5_), as an inflammation-inducing factor, increases the prevalence of lung injury. The aim of this study was to examine the protective effect and mechanism of aerobic exercise on PM_2.5_ exposure-induced lung injury. Forty Wistar rats were randomly divided into four groups: sedentary+PM_2.5_ exposure, exercise+PM_2.5_ exposure, sedentary, and exercise groups. All rats in the exercise-related groups underwent 8-week aerobic interval treadmill training (5daysweek^−1^, 1hday^−1^). PM-exposed rats were exposed to ambient PM_2.5_ (6h day^−1^) for 3weeks after the 8-week exercise intervention. Then, ventilation function, histopathological changes, and inflammation responses of pulmonary tissue were examined. Results showed that PM_2.5_ exposure induced lung injury as manifested by decreased pulmonary function, abnormal histopathological changes, and increased pro-inflammatory cytokine levels (tumor necrosis factor-α and Interleukin-1α). Aerobic exercise alleviated the airway obstruction, reduced respiratory muscle strength, bronchial mucosal exfoliation, ultrastructure damage, and inflammatory responses induced by PM_2.5_ in exercise-related groups. The benefits of exercise were related with the downregulation of p38-mitogen-activated protein kinase (MAPK), and the subsequent inhibition of the pathways of the cyclooxygenase 2 (COX-2) product, prostaglandin E_2_ (PGE_2_). Thus, pre-exercise training may be an effective way to protect against PM_2.5_-induced lung inflammatory injury in rats.

## Introduction

Air pollution, especially fine particulate matters with a diameter less than 2.5μm (PM_2.5_), has become a serious public health problem (Heck et al., 2017; Loxham et al., 2019). The mortality, morbidity, and risk factors in China published in the Lancet showed that particulate matter (PM) pollution is the top fourth risk factor for the number of deaths in 2017 (Zhou et al., 2019). Many epidemiological investigations suggested that PM_2.5_ is associated with respiratory and other non-communicable diseases, and the increase in PM_2.5_ is associated with increased hospital admissions for respiratory diseases (Qiu et al., 2012; Tian et al., 2019). An 18years cohort study indicated that ambient air pollutants was significantly associated with declining lung function and increasing emphysema (Wang et al., 2019a). Additionally, PM_2.5_ exposure could lead to lung injury characterized by pulmonary dysfunction, inflammatory cell infiltration, pulmonary edema, and pulmonary fibrosis (Feng et al., 2019; Li et al., 2019). These harmful effects of PM_2.5_ are related to oxidative stress and inflammatory responses (Li et al., 2018; Zhang et al., 2019). Further researches showed that PM_2.5_ may exacerbate inflammation in murine lung *via* a Toll-like receptor2 (TLR2)/Toll-like receptor4 (TLR4)/MyD88-signaling pathway (He et al., 2017). The activation of TLR4/Nuclear factor Kappa B (NF-κB) observed in PM_2.5_-induced lung epithelial cells could lead to further inflammation infiltration (Gu et al., 2017b). Therefore, it is pivotal to develop therapeutic strategies to prevent the respiratory system injury caused by PM_2.5_.

Regular exercise may be able to modulate the immune system to enhance resistance to respiratory infections (Pedersen and Hoffman-Goetz, 2000; Wang et al., 2020). Numerous researchers have indicated the protective effects of exercise against various diseases through its anti-inflammatory and antioxidant capability (De Sousa et al., 2017; Metsios et al., 2020). A related study reported that moderate-intensity aerobic physical training reduces oxidative stress and protects against the development of emphysema induced by cigarette smoke in mice (Toledo et al., 2012). Our previous work also found that aerobic interval training improves the pulmonary function and impedes the lesion progression induced by acute exposure to different PM_2.5_ concentrations because of the effective inhibition of oxidative stress and inflammation (Qin et al., 2020b). However, the effects of exercise on lung injury induced by sub-chronic exposure to PM_2.5_ are still uncertain, and the underlying mechanism of the protective effects of exercise training remains to be elucidated.

Cyclooxygenase 2 (COX-2) is a well-known inflammatory mediator that can regulate the conversion of arachidonic acid to prostaglandin E_2_ (PGE_2_; Tsai et al., 2017). COX-2/PGE_2_ plays key roles in the pathogenesis of PM_2.5_-induced inflammation (Fernando et al., 2019). PM exposure results in airway inflammation through the upregulation of COX-2/PGE_2_ (Song et al., 2020). PM exposure stimulates COX-2/PGE_2_ inflammatory signaling pathways in human fibroblast-like synoviocytes (Tsai et al., 2017). P38-mitogen-activated protein kinase (MAPK) plays a substantial role in proinflammatory responses and is closely related to the beneficial effect of exercise (Sur et al., 2008). p38-MAPK regulates COX-2 expression (Xu et al., 2008; Yu et al., 2014). Moreover, exercise remarkably inhibits COX-2 activity, which leads to the suppression of pro-inflammatory cytokines (Lee et al., 2015). However, whether p38–COX-2–PGE_2_ signaling pathways are involved in the protective mechanism of exercise against the inflammation induced by PM_2.5_ is unknown. Thus, the inhibition of p38–COX-2–PGE_2_ pathway may provide a preventive approach for the inflammation induced by PM_2.5_.

Here, we used a whole-body inhalation enrichment system to conduct PM_2.5_ exposure toxicology research and exposed rats to real-time PM_2.5_ inhalation for 3weeks. We assessed the pulmonary function, histopathological characteristics, and inflammatory condition of rats to evaluate to whether aerobic interval training plays a protective role against the lung injury induced by PM_2.5_. Additionally, we observed the p38–COX-2–PGE_2_ signaling pathways involved in the protective effect of exercise on ameliorative inflammation. We hypothesized that exercise is related to the downregulated activation of p38-MAPK, which further inhibited the pathways of the COX-2 product, PGE_2_.

## Materials and Methods

### Animals

Male Wistar rats (age: 52±3days, weight: 247±40g) were purchased from Beijing Vital River Laboratory Animal Technology Co., Ltd. All animals were separately raised in a ventilated caging system and exposed to a 12h light – 12h dark cycle (23±1.0°C and 45–55% humidity). All the experimental procedures were approved by the Animal Ethical Committee of China Institute of Sports Science in accordance with the guidelines of experimental animal use (approval number: CISSLA-2017003).

### Experimental Design

**Figure 1:**
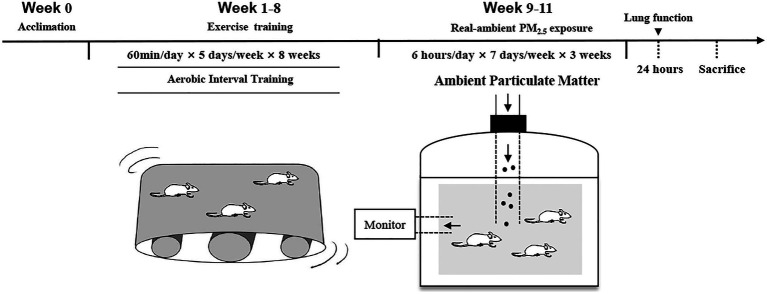
Overview of the experimental procedure.

The animals were randomly assigned to four groups (*n*=10 in each group): sedentary (S), exercise (E), sedentary+PM_2.5_ exposure (S+PM), and exercise+PM_2.5_ exposure (E+PM). All rats in the E-related groups underwent an 8-week aerobic interval treadmill training, and then all rats in the PM-related groups were exposed to PM_2.5_ (Figure 1). The exposure time was set to 6h per day, 7days per week from October 15, 2018 to November 5, 2018, for a total duration of 3weeks. Pulmonary function was examined 24h after the final exposure. Finally, all rats were anesthetized with an intraperitoneal injection and blood was collected through the abdominal aorta. When the rats died, the bronchoalveolar lavage fluid (BALF) and lungs were collected.

### Exercise Program

After treadmill adaptation (1week), animal aerobic interval training was performed with a treadmill (DSPT-202, China) for 1h per time and five times per week for 8weeks. The training protocol consisted of a 5-min warm-up, a 6-min cool-down with 50–55% maximal oxygen uptake (VO_2max_), and seven interval training periods (4min intervals at 80–90% VO_2max_ interspersed with 3min periods of 65–70% VO_2max_). The VO_2max_ was measured through an incremental speed protocol (increased by 5m/min every 3min until the rats were exhausted) with a 0° slope treadmill and Columbus Oxymax Lab Animal Monitoring System (Columbus, United States; Qin et al., 2020a). Then the exercise intensity (80–90% VO_2max_ and 65–70% VO_2max_) corresponding to different treadmill speeds were quantified, respectively.

### PM_2.5_ Exposure System

Rats in PM-related groups were exposed to a PM_2.5_ concentration enrichment system (Beijing Huironghe Technology Co., Ltd., China), which can efficaciously concentrate ambient PM_2.5_ (5–8-folds) without an apparent change in major physicochemical features. The exposure system is located at Zhongguancun Science and Technology Park, Tongzhou District, Beijing, China. The daily air quality index and the concentrations of particulate matters with a particle size below 10μm (PM_10_), sulfur dioxide (SO_2_), nitrogen dioxide (NO_2_), carbon monoxide (CO), and ozone (O_3_) in ambient air are recorded in the website of the Ministry of Ecology and Environment of the People’s Republic of China.^1^ The particle components in the chamber were collected using a 47mm Teflon filter, and the polycyclic aromatic hydrocarbons (PAHs) and metal were analyzed. The PM samples were extracted using a microwave accelerated reaction system and were purified using silica/alumina columns (Ma et al., 2015; Tala and Chantara, 2019). PAHs were analyzed by a gas chromatograph (Agilent 5890A, United States) coupled mass spectrometer (Agilent 5975C, United States). The 16 parent PAHs quantified include naphthalene (NAP), acenaphthene (ACE), acenaphthylene (ACY), fluorene (FLO), phenanthrene (PHE), anthracene (ANT), fluoranthene (FLA), pyrene (PYR), benzo(a)anthracene (BaA), chrysene (CHR), benzo(b)fluoranthene (BbF), benzo(k)fluoranthene (BkF), benzo(a)pyrene (BaP), dibenzo(a,n)anthracene (DanA), indeno (1,2,3-cd) pyrene (IcdP), and benzo(g,h,i)perylene (BghiP). For metal analysis, PM samples were digested using a mixture of HNO3 and HCl (Costas et al., 2010). The concentrations of Cr, Cu, Cd, Ni, Hg, Mn, Zn, Pb, and As were determined using Inductively coupled plasma mass spectrometry (ICP-MS, DIONEX, United States).

### Pulmonary Function Test

Noninvasive small-animal whole-body plethysmography (Buxco, Inc., United States) was performed to detect the pulmonary function of the rats. The test protocol was performed as previously described (Qin et al., 2021). Finally, respiratory dynamics data, including minute ventilation (MV), tidal volume (TV), breathing frequency (*F*), relaxation time (Tr), expiration time (Te), inspiration time (Ti), pause (Pau), 50% expiratory flow (EF50), estimated peak expiratory flow (PEF), and estimated peak inspiratory flow (PIF), were measured and calculated. Pau is a unitless index that estimates bronchoconstriction and is calculated as: Pau=(Te/Tr)^−1^ (Bosnjak et al., 2014).

### Histopathological Analysis

Lung tissues were fixed, embedded in paraffin, and cut into 5μm-thick sections. The tissue sections were deparaffinized, hydrated, stained with hematoxylin and eosin (HE), and then observed by optical light microscopy (100×, 200×, and 400×). An established scoring system that quantify pathological changes in lung tissues during acute lung injury (Matute-Bello et al., 2011; Li et al., 2019) was used, and the test protocol was performed as previously described (Qin et al., 2021).

### Transmission Electron Microscopy Inspection

The lung tissues were immersed in 2.5% glutaraldehyde, then washed with phosphate buffer (PB) solution, and fixed with 1% OsO_4_ for 1h. Afterward, the tissues were washed with PB, then dehydrated in a graded series of ethanol, embedded in araldite, and polymerized for 24h at 60°C. Additionally, ultrathin sections (60nm) were cut and collected on 200-mesh copper grids, stained with lead citrate, and observed with a transmission electron microscope (JEM-2100, Japan).

### Biomarker Estimation

The concentrations of PGE_2_ (Abcam, United States, intra-CV: 5.8%; inter-CV: 5.1%, competitive ELISA), TNF-α (CUSABIO, China, intra-CV: 7.8%; inter-CV: 8.3%, sandwich ELISA), and IL-1α (CUSABIO, China, intra-CV: 7.3%; inter-CV: 7.9%, sandwich ELISA) in BALF supernatants were determined using enzyme-linked immunosorbent assay kits according to the manufacturers’ instructions. Optical density was measured with an enzyme-linked analyzer (MultiskanAsc, Thermo, United States) within 10min. The standard curve was constructed with the standard solution as the ordinate and OD value as the abscissa to help detect the sample concentration.

### Western Blot

Total proteins were extracted from the lung samples by using a RIPA reagent kit. Protein concentrations were determined using the bicinchoninic acid (BCA) method. The proteins were transferred to a nitrocellulose membrane (loading 20μg total protein per gel lane). The membranes for western blot analysis were incubated at 4°C overnight with the following primary antibodies against: Cox-2 (CST, United States; dilution: 1:2000), p38 (CST, United States; dilution: 1:2000), p-p38 (CST, United States; 1:1000), and β-actin (Immunoway, China; 1:500). Secondary antibodies (goat anti-rabbit/mouse IgG, TDYBIO, China, 1:10,000) were added for 40min, and then the membranes were washed with TBST. Finally, immunoreactive bands were detected with an enhanced chemiluminescence (ECL) kit, and band density was analyzed by ImageJ software (National Institutes of Health, United States).

### Statistical Analysis

All data are expressed as mean±SD. Statistical analysis was performed using SPSS software (version 22.0, IBM SPSS Statistics, Chicago, IL, United States). The normality of data distribution was confirmed by Shapiro–Wilk test. Two-way ANOVA (PM_2.5_ and exercise as factors) were used to compare the differences between groups. *p*<0.05 was considered significant. In addition, effect size estimates (Cohen’s d) were calculated to assess and categorize efficacy as small (*d*=0.2), medium (*d*=0.5), or large (*d*=0.8; Lakens, 2013).

## Results

### PM_2.5_ Concentration and Composition

During the 3weeks of PM_2.5_ exposure, the average PM_2.5_ concentrations inside the concentrated PM_2.5_ chamber was 237.01±206.41μgm^−3^, the maximum concentration of 651±70μgm^−3^, and the minimum concentration was 21±41μgm^−3^. The daily PM_2.5_ concentration inside the chamber exceeded 150μgm^−3^ for 11days from October 15, 2018 to November 5, 2018. The averages of other air components (SO_2_, NO_2_, CO, O_3_, and PM_10_) were not remarkably different between inside and outside the chambers. The concentrations of PAHs and metal inside the chamber are shown in Table 1. Phenanthrene was the most prevalent PAH, followed by fluorene and acenaphthene, and the top three prevalent metallic elements were Zn, Mn, and Cu.

**Table 1:** Average mass concentration of metals and polycyclic aromatic hydrocarbons (PAHs) in PM_2.5_.

Metals	Mass concentration (ngm^−3^ PM_2.5_)	PAHs	Mass concentration (μgm^−3^ PM_2.5_)
Cr	11.41	Naphthalene	0.127
Mn	68.15	Acenaphthylene	0.033
Ni	4.80	Acenaphthene	1.108
Cu	21.91	Fluorene	1.145
Zn	216.38	Phenanthrene	2.195
As	12.32	Anthracene	0.145
Cd	2.94	Pyrene	0.293
		Fluoranthene	0.183
		Chrysene	0.052
		Benzo(a)anthracene	0.115
		Benzo(b)fluoranthene	0.207
		Benzo(k)fluoranthene	0.067
		Benzo(a)pyrene	0.102
		benzo(g,h,i)perylene	0.148
		Indeno(1,2,3,c,d)pyrene	0.153
		Dibenz(a,h)anthracene	0.052

### PM_2.5_ Exposure Decreased Lung Pulmonary Function, and Exercise Played a Protective Role in This Process

**Figure 2:**
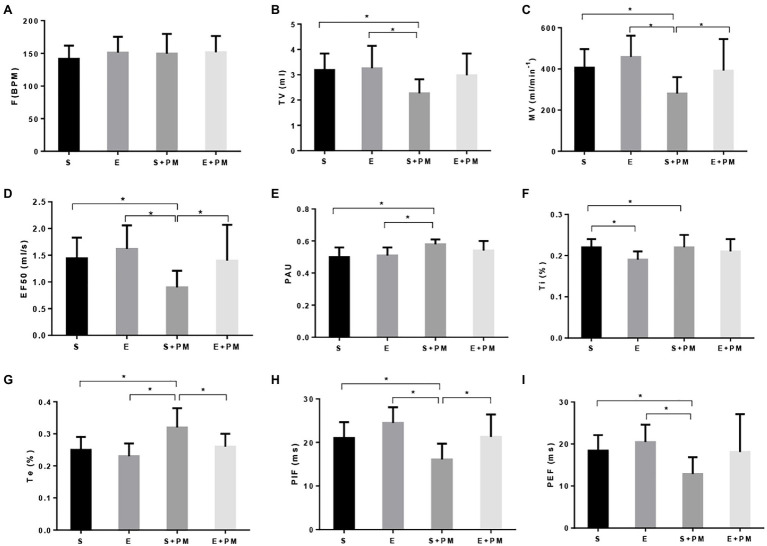
Aerobic exercise protected rats from PM_2.5_-induced pulmonary function decrease. **(A)** Breathing frequency (*F*). **(B)** Tidal volume (TV). **(C)** Minute ventilation (MV). **(D)** Forced expiratory flow-50% (EF50). **(E)** Pause (PAU). **(F)** Inspiration time (Ti). **(G)** Expiration time (Te). **(H)** Estimated peak inspiratory flow (PIF). **(I)** Estimated peak expiratory flow (PEF). Data represent mean±SD. ^*^p<0.05 between groups.

WBP was performed to evaluate the pulmonary function of rats. The F (Figure 2A) and Ti (Figure 2F) had no significant change in the S+PM2.5 compared with the S group. Obvious reduction in TV (*p*<0.05; ES=1.52, Figure 2B), MV (*p*<0.05; ES=1.48, Figure 2C), EF50 (*p*<0.05; ES=1.53, Figure 2D), PIF (*p*<0.05; ES=1.35, Figure 2H), and PEF (*p*<0.05; ES=1.45, Figure 2I) was observed in the S+PM_2.5_ group compared with the S group after 3weeks of PM_2.5_ exposure. Significant increase in PAU (*p*<0.05; ES=1.69, Figure 2E) and Te (*p*<0.05; ES=1.37, Figure 2G) were found in the S+PM_2.5_ group compared with the S group. These results indicated that PM_2.5_ induced a decrease in pulmonary ventilation function (TV and MV), promoted tract obstruction (EF50, PAU, and Te), and weakened respiratory muscle strength (PIF and PEF).

The TV (*p*>0.05; ES=0.98, Figure 2B), MV (*p*<0.05; ES=0.91, Figure 2C), EF50 (*p*<0.05; ES=0.96, Figure 2D), PIF (*p*<0.05; ES=1.17, Figure 2H), and PEF (*p*>0.05; ES=0.75, Figure 2I) of E+PM rats were promoted compared with those of the S+PM rats after 8weeks of aerobic pre-exercise. Moreover, the PAU (*p*>0.05; ES=0.84, Figure 2E) and Te (*p*<0.05; ES=1.18, Figure 2G) of the S+PM_2.5_ group significantly declined compared with those in the S group. These results indicated that 8weeks of aerobic interval training alleviated the pulmonary dysfunction caused by PM_2.5_ in rats, especially pulmonary ventilation function, tract obstruction, and respiratory muscle strength.

### Exercise and PM_2.5_ Exposure Pathologically Affect the Characteristics of Lung Tissue

**Figure 3:**
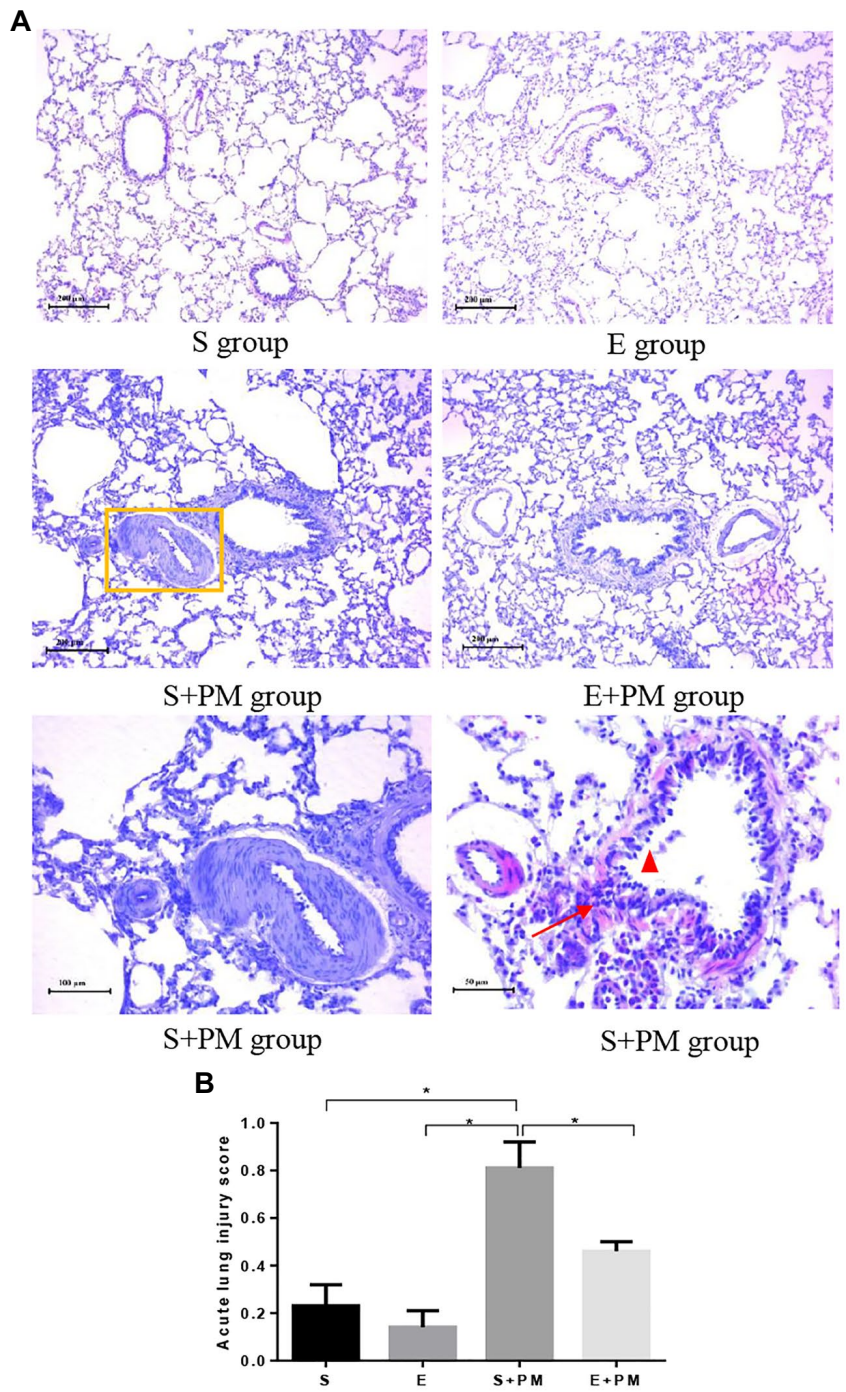
Photomicrographs of rat lung (HE stain) in different groups. **(A)** Histologic analysis of lung tissues. Yellow rectangle indicated pulmonary artery; red triangle indicated mucous membrane exfoliation; red arrows indicated inflammatory infiltration. **(B)** Acute lung injury scores in different groups. Data represent mean±SD. ^*^p<0.05 between groups.

Morphological alterations in lung tissues were evaluated by HE staining as shown in Figure 3. Lung structures were almost normal in the S and E groups (Figure 3A). PM_2.5_ exposure led to peribronchiolar neutrophil infiltration (Figure 3A), alveolar septal thickening (Figure 3A), bronchial mucosal exfoliation and lesions (Figure 3A), pulmonary arterial smooth muscle hypertrophy, and pulmonary vascular lumen stenosis (Figure 3A). Inflammatory infiltration and the degree of lesions in the peribronchiolar and vascular walls were ameliorated in the E+PM group compared with those in the S+PM_2.5_ group (Figure 3A). Meanwhile, the lung injury score of the E+PM_2.5_ group was significantly lower than that in the S+PM_2.5_ group (*p*<0.05; ES=4.23, Figure 3B), which means that the 8-week aerobic pre-exercise program has a preventive effect against lung tissue injury.

**Figure 4:**
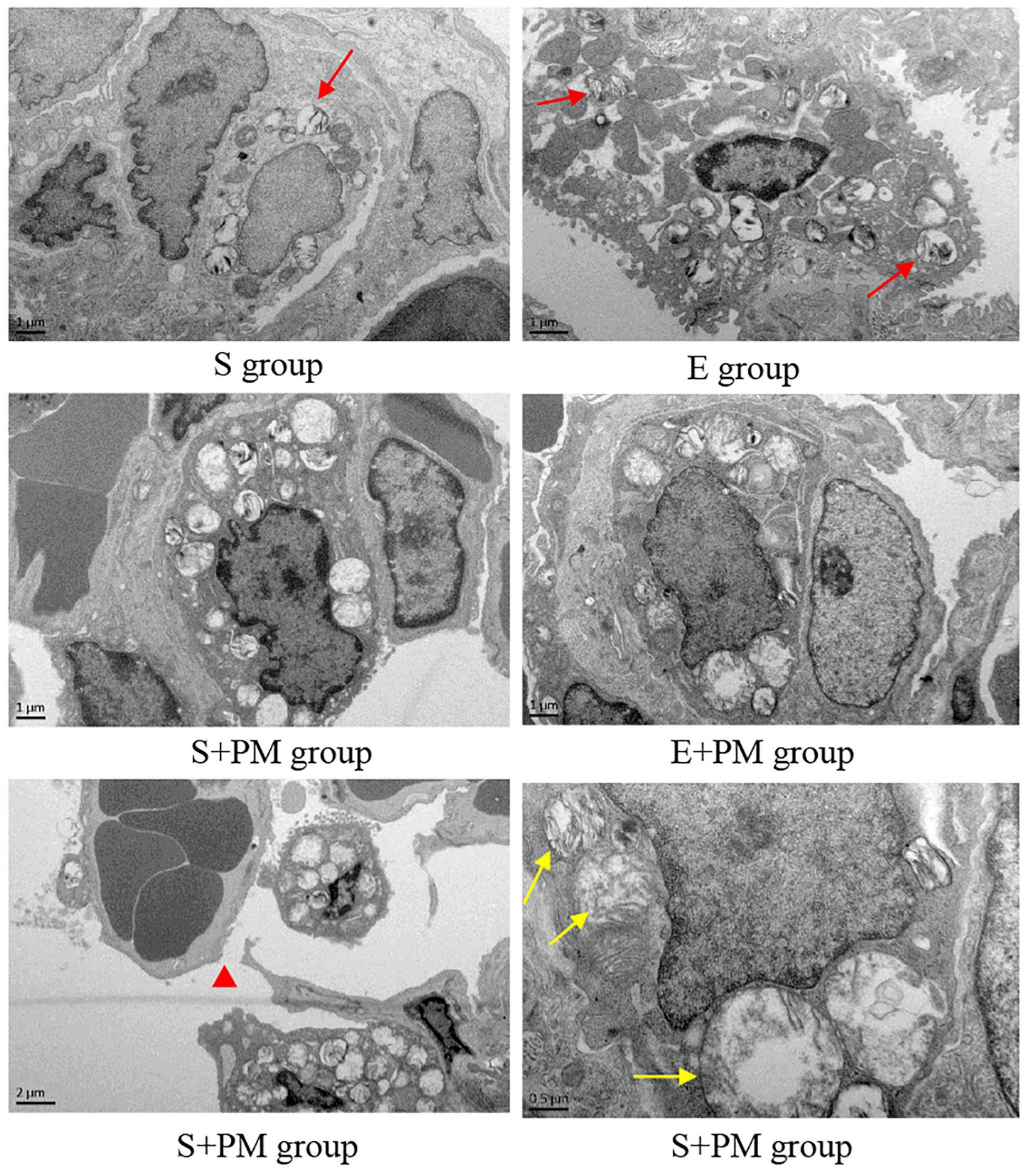
Electron micrograph of lung tissue in different groups. Red arrows indicated lamellar bodies; red triangle indicated Basement membrane cracked after PM_2.5_ exposure; yellow arrows indicated mitochondrial swelling and vacuolization in the S+PM group.

Furthermore, the ultrastructure of rat lung tissues was observed. Normal lung epithelial cells (type I and type II alveolar cells) and the tissue matrix of lung interval were observed in the S and E groups (Figure 4). However, more lamellar bodies and microvilli were found in the type II alveolar cells in the E group (Figure 4) compared with the S group (Figure 4). After 3-week PM_2.5_ exposure, the ultrastructure of type II alveolar cells indicated obvious injury, such as mitochondrial swelling and vacuolization (Figure 4) and microvilli reduction or shedding (Figure 4). A part of the lumen was necrotic, and the basement membrane was fractured in a large area (Figure 4). Compared with the S+PM_2.5_ group, the lamellar bodies in type II alveolar cells increased considerably and the degree of mitochondrial injury improved in E+PM_2.5_ rats (Figure 4).

### Exercise Suppressed PM_2.5_-Induced Inflammation in the Lung

**Figure 5:**
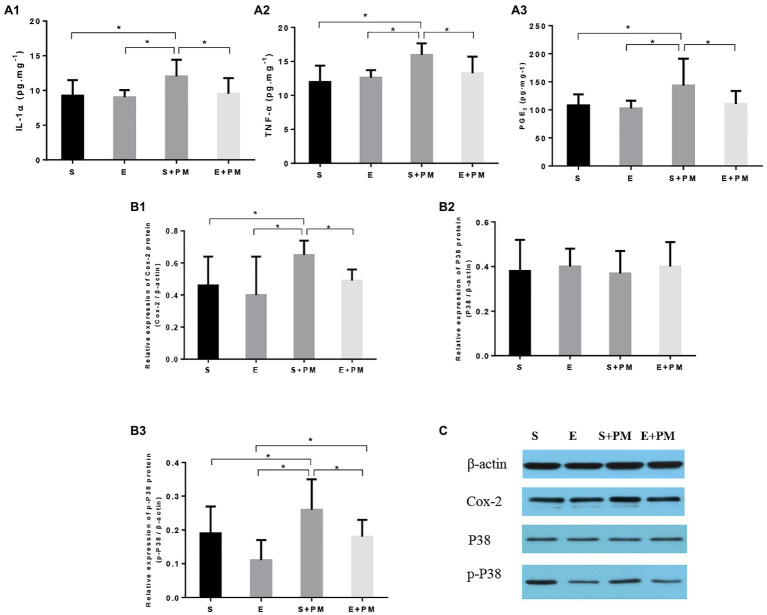
Aerobic exercise suppressed PM_2.5_-induced lung inflammation in rats. **(A1–A3)** Pro-inflammatory cytokines (IL-1α and TNF-α) and PGE_2_ in bronchoalveolar lavage fluid (BALF). **(B1–B3)** Protein levels of Cox-2, p38, and p-p38 in the lung tissues of rats. **(C)** Protein binding pattern determined by Western blot analysis. Data represent mean±SD. ^*^p<0.05 between groups.

We examined proinflammatory cytokines in the BALF to characterize the inflammatory response *in vivo*. As shown in Figures 5A1,A2, the levels of IL-1α (*p*<0.05; ES=1.19) and TNF-α (*p*<0.05; ES=1.92) obviously increased in S+PM rats compared with those in S rats. Moreover, aerobic interval training resulted in a significant downregulation in the levels of TNF-α (*p*<0.05; ES=0.53) and IL-1α (*p*<0.05; ES=1.07) compared with those in the S+PM group.

The p38–COX-2–PGE_2_ pathway is closely related to the pro-inflammatory effects of PM_2.5_ exposure and the anti-inflammatory activity induced by exercise. Therefore, we further examined the expression levels of proteins related to the p38–COX-2–PGE_2_ pathway (Figures 5A3,B1–B3. The protein levels of PGE_2_ (*p*<0.05; ES=0.98, Figure 5A3), COX-2 (*p*<0.05; ES=1.34, Figure 5B1), and p-p38 (*p*<0.05; ES=0.75, Figure 5B3) showed an obvious increase in the S+PM group than in the S group after 3weeks of PM_2.5_ exposure. Apparently, the levels of PGE_2_ (*p*<0.05; ES=0.89, Figure 5A3), COX-2 (*p*<0.05; ES=1.98, Figure 5B1), and p-p38 (*p*<0.05; ES=1.09, Figure 5B3) were effectively downregulated in the E+PM group compared with those in the S+PM group. The protein binding pattern determined by Western blot analysis were presented in Figure 5C.

Overall, aerobic exercise resulted in a remarkable decrease in pro-inflammatory cytokines and inflammatory pathway-related proteins. The results indicated that 8-week aerobic interval training could inhibit the inflammation induced by PM_2.5_.

## Discussion

The major findings of the present study are that aerobic interval training may be an effective way to protect against PM_2.5_-induced lung inflammation in rats, and the p38-COX-2-PGE2 signaling pathways might be involved in the protective effect of exercise on ameliorative inflammation. These findings implied that regular exercise training can effectively improve immune function, especially for the young individuals with high amounts of sedentary behavior. Therefore, developing regular exercise habits is essential for the young individuals to against the injury of smog weather. Our study suggests that exercise training may be as an adjuvant in the prevention of pulmonary disease due to PM_2.5_ exposure.

In this study, a whole-body inhalation PM_2.5_ enrichment system for rats was used to mimic the actual living environment of humans to the greatest extent possible (Chu et al., 2019; Su et al., 2020). The mean mass concentration of exposure chambers in our study was 237.01±206.41μgm^−3^, which is a relatively moderate pollution levels of air pollution according to the Air Quality Guidelines of China. The mean mass concentration of PM_2.5_ represented the ambient air pollution in early winter in northern China. In our animal models, after 3weeks subacute PM_2.5_ exposure, the significant airway obstruction, declined of respiratory muscle strength, bronchial mucosal exfoliation, stenosis of the pulmonary vascular lumen, ultrastructure damages (mitochondrial swelling and microvilli shedding) were observed in S+PM_2.5_ rats compared with the control. In a previous study, after 89.95μgm^−3^ PM_2.5_ exposure for 3weeks, there was increasingly inflammation characterized by alveolar capillary congestion and peribronchiolar neutrophils infiltration in mice (Li et al., 2019). Yang et al. (2018) reported that 2-week (750μgm^−3^, 4h day^−1^, 5daysweek^−1^) PM_2.5_ inhalation results in lung edema and lesions, oxidative stress, and acute inflammatory responses, which subsequently result in lung tissue damage. The main factors of PM_2.5_-induced lung injury are related to the concentration, duration, and composition of PM_2.5_ (Ning et al., 2019). Moreover, in previous study, we observed a low tolerance of aging rats (16months vs. 8weeks) to high concentrations of PM_2.5_. 2-week exposure duration of whole-body PM_2.5_ inhalation led to lung injury, and the degree of lung injury in aging rats were more deleterious than that of present result in young adult rats (Qin et al., 2021). Meanwhile, the PM_2.5_ exposure duration in aging rats were less than that of young adult rats (4h/day; 2weeks vs. 6h/day; 3weeks). It is also implied that aging individuals are susceptible to PM_2.5_-induced lung injury.

Furthermore, we also analyzed the composition of PM_2.5_ inside the exposure chambers. The prevalent toxic heavy metals, including Zn and Cr, and the common PAHs, including phenanthrene, can activate oxidative stress and inflammation in lung tissues (Maret, 2012; Han et al., 2019; Ma et al., 2020). Growing evidence suggests that these toxic elements in PM_2.5_ are closely related to the development of lung injury (Ning et al., 2019; Qin et al., 2021). In addition, heavy metals, including Zn, Cu, and Cr, and PAHs, including acenaphthylene and phenanthrene, are associated with diesel and gasoline exhaust emissions (Valavanidis et al., 2006; Hu et al., 2016). Due to the exposure chambers of present study located near several high-speed road and urban trunk road in Tongzhou, Beijing, the primary point sources of ambient PM_2.5_ were derived from motor vehicle exhaust, which further implied that traffic air pollution related to lung health. Thus, we should try to avoid rush-hour traffic when smog condition is serious.

Proverbially, physical activity is an effective nonpharmacological treatment for the prevention of chronic diseases and the enhancement of immunity; moreover, exercise type, intensity, and duration are the main factors that impact the intervention effect (MacInnis and Gibala, 2017; Nieman and Pence, 2020). In the present work, we selected aerobic interval training. Aerobic interval training can provide cardiorespiratory fitness and body fat reduction that are similar to or greater than those of traditional moderate-intensity continuous training (Molmen-Hansen et al., 2012; Wewege et al., 2017). Moreover, aerobic interval training has changeable exercise rhythm and challenging intensity, therefore, it has become an attractive approach for adults. In addition, we measured the VO_2max_ of rats to ensure an accurate initial exercise intensity, which was adjusted every 2weeks to maintain satisfactory exercise effects (Qin et al., 2020a). Our results proved that the 8-week aerobic interval training alleviated the declined in ventilation function and respiratory muscle strength, as well as released tract obstruction; these effects may be related to the improvement of peribronchiolar and vascular wall lesions and inflammatory infiltration after regular exercise training. The findings are directly in line with previous findings (Rietberg et al., 2017). Notably, lamellar bodies and microvilli in type II alveolar cells increased after the 8-week aerobic interval training. Lamellar bodies are the specialized secretory organelles of type II alveolar cell that package phospholipid film and regulate its secretion (Mulugeta et al., 2002). The phospholipid film of dipalmitoyl phosphatidylcholine on the surface of lung alveoli reduces surface tension for optimal gas exchange and builds a hydrophobic protective lining as environmental barrier (Schmitz and Müller, 1991; Menon et al., 2018). One study indicated that lamellar bodies in A549 cells disappear compared with the control when exposed to 100μgPM_2.5_ (Peng et al., 2019). A similar change was also observed in our studies. However, research on the direct effect of exercise training on lamellar bodies is rare. Our results showed that the increase in lamellar bodies may be associated with the improvement of pulmonary ventilation and compliance function after the 8-week aerobic exercise training. Our research also provided direct *in vivo* evidence that the protective effects of aerobic interval exercise mediated the pulmonary dysfunction induced by PM_2.5_ exposure.

The lung injury induced by PM_2.5_ is linked to inflammatory responses (Feng et al., 2019), including peribronchiolar neutrophil infiltration, increased proinflammatory cytokines, and the activation of relative inflammatory pathway. Zhang et al. (2019) found that PM_2.5_ induced Rac1and regulated AKT signaling associated with lung inflammation (Zhang et al., 2019). Gu et al. (2017a) indicated that PM_2.5_ promotes the overactivation of the Notch signaling pathway and aggravates the immune disorder of COPD. Gu et al. (2017b) reported that PM_2.5_ induced lung epithelial cells by the activation of TLR4/NF-kB leading to inflammation infiltration. Thus, PM_2.5_ induced inflammatory responses may be an essential factor of lung injury. Similar results were obtained in present study. In addition, previous studies also investigated the effect of intermittent exercise on immune function. Interval training could modulate autoimmunity by decreasing the polarization of T cells into deleterious Th1 and Th17 cells (Goldberg et al., 2021). Ten weeks of low-volume, high-intensity interval exercise could improve neutrophil and monocyte function and enhance innate immune system in sedentary adults (Bartlett et al., 2017). Animal research also indicated that interval exercise training could reduce the inflammation induced by cisplatin nephrotoxicity and downregulate the TLR4/NF-κB signaling pathway (Leite et al., 2021). In the present study, we verified that aerobic interval training produces similar anti-inflammatory effects for PM_2.5_-induced inflammation. Furthermore, p38–COX-2–PGE_2_ signaling pathways were assessed to explore the putative mechanisms of protective effect of exercise against the lung injury induced by PM_2.5_ exposure. After 3weeks of PM_2.5_ exposure, the high expression of p-p38, COX-2, and PGE_2_ presented inflammatory response as induced by PM_2.5_. However, we found that aerobic interval training could prevent the rise in p-p38 level and then hindered the activation of the COX-2-PGE_2_ inflammatory pathway. Consequently, our data indicated that exercise could alleviate the lung injury induced by PM_2.5_ possibly through p38–COX-2–PGE_2_ pathway. Currently, the detailed mechanisms of the p38–COX-2–PGE_2_ pathway involved in inflammation prevention by exercise is still uncertain and needs further verification.

This study has several limitations. First, we only used male rats. The deleterious effects of PM_2.5_ and physiological characteristics are different between sexes (Kampa and Castanas, 2008; Wang et al., 2019b). However, we only selected male rats without considering the estrous cycle. Second, only the protective effects of exercise on lung injury induced by subacute PM exposure (3weeks) were observed. Whether the aerobic exercise training has a protective effect on long-term exposure to smog weather will be studied in the future. Lastly, the detailed mechanisms of the p38–COX-2–PGE_2_ pathway in preventing inflammation needs additional analysis. The critical regulatory molecules underlying these interactive effects warrant further investigation.

In summary, we showed that 8-week aerobic interval exercise may be an effective way to protect against PM_2.5_-induced lung inflammation in rats. Aerobic exercise alleviated the airway obstruction, weakened respiratory muscle strength, bronchial mucosal exfoliation, ultrastructure damage, and inflammatory responses induced by PM_2.5_ in exercise-related rats. These benefits of exercise were related with the downregulated activation of p38 and MAPK, which further inhibited COX-2-PGE_2_ pathways. In future, the guidelines in different age groups on exercise promotion behavior combined with environmental factors are essential. It requires further researches to elucidate the relevant mechanism of exercise on preventing the injury induced by PM_2.5_ exposure, which will offer an effective measure for health promotion and diseases prevention.

## Data Availability Statement

The original contributions presented in the study are included in the article/supplementary material; further inquiries can be directed to the corresponding author.

## Ethics Statement

The animal study was reviewed and approved by Animal Ethical Committee of the China Institute of Sports Science.

## Author Contributions

FQ, ZF, and MX contributed equally in the ideas, writing of the manuscript, and drafted the manuscript. FQ and JZ conceived and designed the research and edited and revised the manuscript. MX, ZF, ZW, YD, and FQ performed the experiments. FQ, SC, and ZF analyzed the data. FQ, JZ, and CQ interpreted the experimental results. CQ, SC, and LZ prepared the figures. JZ approved the final version of the manuscript. All authors contributed to the article and approved the submitted version.

## Funding

This work is supported by the China Postdoctoral Science Foundation (2018T110076), the National Natural Science Foundation of China (31900845 and 11775059), and the Fundamental Research Foundation of the China Institute of Sport Science (20-18).

## Conflict of Interest

The authors declare that the research was conducted in the absence of any commercial or financial relationships that could be construed as a potential conflict of interest.

## Publisher’s Note

All claims expressed in this article are solely those of the authors and do not necessarily represent those of their affiliated organizations, or those of the publisher, the editors and the reviewers. Any product that may be evaluated in this article, or claim that may be made by its manufacturer, is not guaranteed or endorsed by the publisher.

## Footnotes

^1^ https://air.cnemc.cn:18007/
